# Experimental Technique to Study the Interaction Between a Bubble and the Particle-Laden Interface

**DOI:** 10.3389/fchem.2018.00348

**Published:** 2018-08-14

**Authors:** Xingshi Yang, Alexander Mayer, Ghislain Bournival, Robert Pugh, Seher Ata

**Affiliations:** ^1^School of Mining Engineering, University of New South Wales, Sydney, NSW, Australia; ^2^Department of Physics and Mathematics, Nottingham Trent University, Nottingham, United Kingdom

**Keywords:** particle monolayer, packing factor, particle tracking, surface pressure, bubble coalescence

## Abstract

An experimental apparatus was developed based on the Langmuir-Blodgett trough design to investigate the compression of monolayers of micron size spherical glass particles at the air-water interface and the interaction of an air bubble with the monolayers. The setup modifies the regular Langmuir-Blodgett trough by using a deep and clear glass cell. The cell allowed both the optical observation of the particle monolayer and the insertion of a capillary to produce a bubble under the layer of particles. Surface pressure-area (Π-A) isotherms were measured while the particles rearranged at the interface during compression and expansion for different pH values and particle wettability. We also analyzed the motion of particles in the monolayer by the surface pressure and packing factor to gain further insights into the behavior of particles during the coalescence process. The results suggested that the coalescence of a bubble was dependent on the formation of a defect in the particle layer and the defect size was both strongly influenced by particle hydrophobicity and the pH of the subphase.

## Introduction

Langmuir monolayers and Langmuir–Blodgett (LB) films are widely used to investigate the morphology and dynamics of particle monolayers at air-water interfaces. Most studies focus on insoluble surfactant layers and nanoparticles owing to their wide range of applications, but there have been several investigations of visible micron size particles with different surface wettabilities (Santini et al., 2011; Kralchevsky et al., 2016; Petkov et al., 2016). Hórvölgyi et al. (1996) investigated the behavior of a monolayer consisting of artificially hydrophobized glass beads of 75 μm at the air-water interface in a Langmuir film balance and found that the reorganization occurring in the particle-laden interface was strongly correlated to the particle hydrophobicity and surface packing density. Upon mechanical compression of the interface, particle layers of high density were found to buckle into bilayer structures and/or collapse. It was also found that the energy required to compress the most strongly hydrophobic particles into a close-packed layer was significantly greater than that required for the compression of the less hydrophobic particles. Onoda (1985) studied the assembly of polystyrene particles in the size range of 2–15 μm at a flat air-water interface and found that particle clustering was strongly dependent on the particle size. For 2 μm particles, the particle rafts appeared to be well ordered while the clusters formed from large particles displayed less stable and loose agglomerates with branched structures.

The formation and ordering of particles at an interface are complex phenomena and the physics governing the process are controlled by various factors such as particle size and shape, nature of the phases forming the interface and hydrophobicity of particle material. In recent years, several studies have combined the Langmuir trough technique with surface-specific analysis techniques to provide further insights into the complex behavior of particles at liquid interfaces. Some examples of such techniques include x-ray diffraction (Yun and Bloch, 1989; Fujii et al., 2017), grazing incidence x-ray diffraction (Reitzel et al., 2000), ellipsometry (Hunter et al., 2009), Brewster angle microscopy measurements (Safouane et al., 2007), vibrational sum frequency generation spectroscopy (Ma and Allen, 2006), atomic force microscopy (Reitzel et al., 2000) and imaging techniques such as microscopy and high speed camera (Cote et al., 2009; McNamee et al., 2011). There have also been studies where the Langmuir-Blodgett trough was modified to examine the orientational order, packing, and morphology in monolayers or the behavior of the monolayer as a whole. Krägel et al. (1996) used a modified Langmuir-Blodgett trough with an oscillating barrier to generate periodic dilation and compression to measure the dilational elastic modulus as a function of surface area. The method permitted a direct measurement of the amplitude of surface pressure oscillation and the phase angle between the generated area oscillation and the resulting pressure oscillations. A similar technique was used by Planchette et al. (2013) where the air-water interfaces coated with monodispersed hydrophobic silica particles with diameters ranging from 35 to 159 μm were oscillated with a vertical oscillating glass plate coupled to a vibrating pot and frequency generator. The mechanical properties of particle-laden interfaces were investigated by studying capillary wave propagation along the interface.

Langmuir monolayers provide an excellent model for studying the properties of the surface layers in response to compression and shear. Such studies are important in understanding the behavior of particles at an air-bubble surface in a turbulent environment. Particle coated bubbles are found in a number of processes and applications, such as froth flotation. In mineral flotation, air bubbles in the range of 1 mm are introduced into water containing suspended solid particles with various surface hydrophobicities in a suitable flotation vessel. Hydrophobic particles attach to the surface of bubbles and rise to the surface of the liquid where they form a froth layer, which overflows the lip of the vessel. For a particle to attach to a bubble, both must first be brought together, generally in a highly turbulent environment. Particle coated bubbles trapped in turbulent flow undergo intensive surface deformation with large amplitude shape oscillation (Schulze, 1984) leading to compression and expansion of the particle layer in response to the shear applied. Similarly, bubbles in the froth layer undergo surface deformation as a result of continuous rearrangement and morphological changes caused by bubble coalescence within the froth (e.g., Pugh, 1996; Bournival et al., 2015a), bubble bursting on the top layer (Barbian et al., 2003), and drainage of water (Pugh, 1996). Understanding the behavior of the particle layer at bubble surfaces is important for the fine tuning of bubble stability and for optimizing the efficiency of froth flotation as well as other processes that include particle coated bubbles and foams.

The aim of this study is to introduce an experimental method based on the Langmuir-Blodgett trough. It consists of a trough modified with a transparent deep glass cell, which made it possible to produce an air bubble through a capillary tube beneath the particle monolayer. As demonstrated in this study, the setup allowed direct observation of interaction between a bare bubble and a particle-laden interface, while also being capable of controlling the particle coverage at the interface. By analyzing the recordings during the interaction of the bubble and the particles, the changes in the particle packing and particle movement could be quantified and related to the stability of the interface. Two-dimensional particle networks were studied in order to investigate their ability to stabilize bubbles. This paper shows the different analyses, which can be conducted with the experimental setup. It is part of a larger body of work, which will examine the interaction of surfactants and particles with bubbles.

## Experimental procedure

### Materials

Soda-lime glass beads with a density of 2.5 g/cm^3^ were purchased from Potters Industries Pty Ltd. (Melbourne, Australia). The particles had a volume-surface equivalent diameter (Sauter diameter, *D*_32_) of 64 μm while the 90% passing diameter (*D*_90_) was *D*_90_ = 92 μm. While most of the particles were nearly spherical in shape, some irregularly shaped particles were also found. The glass particles were cleaned in a mixture of ammonia (Ajax Finechem Pty Ltd., 28%) and hydrogen peroxide (Chem-Supply, 30% w/w) and hydrophobized with 1-octanol (Sigma-Aldrich, 99%) or 1-butanol (Chem-Supply, 99%) as described below. Dichlorodimethylsilane (DCDMS) (Sigma-Aldrich, ≥99.5%) and hydrochloric acid (HCl) (Chem-Supply, 32% w/w) were employed in the hydrophobization of glass plates making up the trough. All glassware was cleaned in a mixture of ethanol and sodium hydroxide (Sigma-Aldrich, ≥97.0%).

During the experiments, the pH of the aqueous phase was adjusted with hydrochloric acid or sodium hydroxide and the ionic strength of solution was kept at 0.01 M with sodium chloride (Ajax Finechem Pty Ltd., analytical grade). The spreading solvent for particle dispersion was spectroscopic grade toluene (Chem-Supply, ≥99.5%). All water used throughout was Milli-Q water from a Millipore system. The water had a resistivity of 18.2 MΩ cm and a surface tension of 72.8 mN/m. All experiments were conducted in a temperature controlled room (22 ± 1.5°C).

### Cleaning and hydrophobization methods

Glass beads used in the experiments were cleaned in an alkaline solution to remove any organic surface films through an oxidative breakdown of the contaminants following the procedure of Ata (2009). A solution of 50 mL of ammonia in 250 mL of water was heated to 80°C. A total of 50 g of particles was added to the hot solution with 50 mL of hydrogen peroxide. The mixture was stirred for 5 min and left to cool down to room temperature. The particles were decanted and rinsed with Milli-Q water until the pH reached that of Milli-Q water. The particles were oven-dried at 60°C, then transferred to a desiccator to minimize contact with moisture.

Particles were hydrophobized by esterification following the method of Bournival et al. (2015b). A total weight of 130 g of 1-octanol or 1-butanol was refluxed in a conical flask with 50 g particles for 7 h to produce a surface of intermediate hydrophobicity. The equilibrium contact angles in the water phase (i.e., at natural pH) were 75°For the 1-octanol treated particles and 43°For the 1-butanol treated particles (Bournival et al., 2014). The particles are referred to as more hydrophobic (75°) or less hydrophobic (43°) throughout. The particle-alcohol mixture was then cooled down to room temperature and the alcohol decanted. The particles were twice suspended in acetone and ethanol to remove the excess alcohol. The particles were dried in the oven at 40°C and stored in a desiccator. It should be noted that the contact angle is expected to slightly change with the pH of the subphase, with a decrease in the contact angle at elevated pH (i.e., about 9) as demonstrated by Laskowski and Kitchener (1969). However, more work is needed to determine the effect of the contact of the spreading solvent with the hydrophobized surface on the contact angle. The values of the contact angles were assumed to be equivalent to that reported in the literature.

Glass plates, which made up the trough, were first placed in an ethanol-sodium hydroxide solution following the procedure described by Bournival et al. (2015b) and then dried in an oven at 40°C. Once dried, the plates were protonated by soaking in a 5% (v/v) HCl solution and rinsed with Milli-Q water prior to drying in an oven at 90°C for 30 min. The protonation helps the silanation process by forming head groups which are important for the reaction of the DCDMS with the surface. Once the plates cooled down to 35°C, they were placed in a desiccator along with a small beaker containing 3 mL of DCDMS for 3 h to expose the glass surface to silane vapor. The glass substrates were then baked in the oven at 125°C for 30 min, cooled down to room temperature, and sonicated in toluene to remove any residual (unbound) molecules and polymer. The hydrophobized glass plates had a contact angle of 90 ± 5° in water as measured by the static sessile drop method.

### Experimental setup

**Figure 1:**
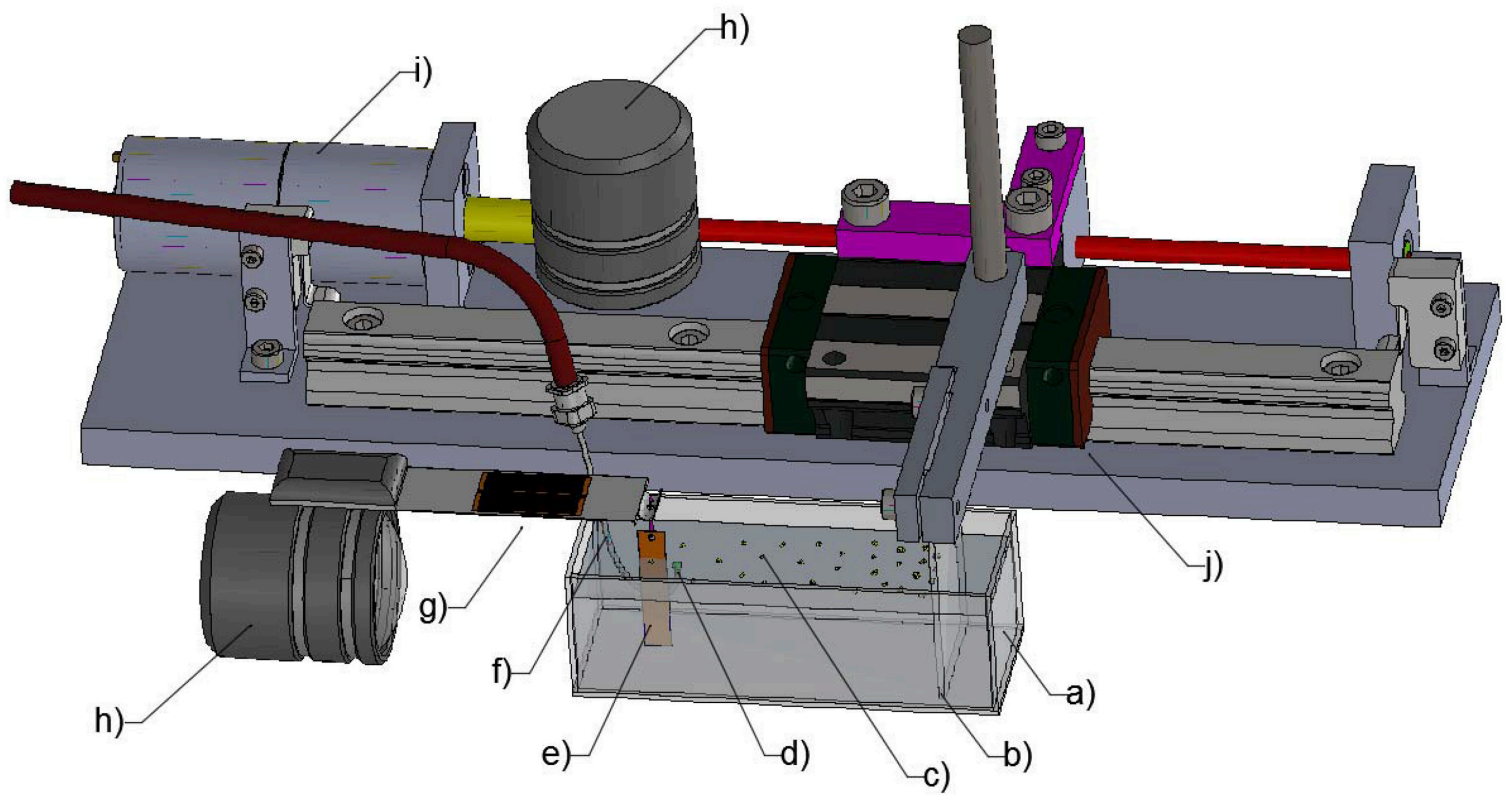
Schematic of the modified Langmuir-Blodgett trough: **(a)** transparent glass cell; **(b)** barrier; **(c)** particle-laden interface; **(d)** bubble; **(e)** Wilhelmy plate; **(f)** the capillary; **(g)** the loadcell; **(h)** high-speed camera; **(i)** motor; **(j)** rail carriage block.

An experimental setup was built to study the interaction between individual particles positioned at an air-water interface while subjecting the interface to compressive, expansive, and oscillatory (i.e., bubble coalescence induced) forces. The surface is considered representative of the air-water interface found at a bubble's boundary. The setup was modified from the Langmuir-Blodgett trough as shown in Figure 1. The cell (75 × 25 × 25 mm) was made of hydrophobized glass plates. The projected area (area of the particle-laden interface under compression) ranged from 500 to 1,750 mm^2^. A motor (Uxcell, DC 6 V) rotated the screw that drove the rail carriage block (HIWIN, HGW15CC) to move linearly along the track. A hydrophobized glass plate (25 × 40 mm) was used as a barrier which was driven by the rail carriage block. The area of the particle-laden interface was controlled by the moving barrier at a rate of 11.25 mm^2^/s with a barrier moving speed of 0.45 mm/s or a rate of 20 mm^2^/s for a moving speed of 0.8 mm/s for some experiments (section The Effect of Particle Shape and Size on Particle Displacement During Compression). The Wilhelmy plate, made of a mica sheet (TED PELLA, grade V1, 5 × 25 mm), was hung on a loadcell perpendicular to the moving barrier. The loadcell used for the force measurement was made from four micro strain gauges (#CEA-06-500UW-120) in full bridge configuration (one gauge per leg) and had the precision of 0.1 mg. The instrument was connected to a National Instrument system and recorded the data in Labview every 10 ms. A hook-shaped stainless steel capillary (inner diamater = 0.69 mm, outer diameter = 1.04 mm) was connected to a syringe mounted on an Aladdin® Syringe Pump in order to create an air bubble just beneath the air-water interface. The distance between the capillary tip and the interface was maintained at 2 mm in all experiments. The whole setup sat on a vibration-free table to eliminate unwanted surface vibrations.

The images of the bubble and its interection with particle monolayers were captured on a high-speed camera (Photron FASTCAM APX RS, Photron USA) from both the top and side views. The top view, for which results are presented in this study, used a long distance lens (DistaMax, Infinity Photo-Optical) covering an area of 3.4 × 4.5 mm. The compression and expansion of the particle layers were recorded at a capture rate of 50 frames per second (fps) while the oscillation of the interface following the coalescence of a bubble with the interface was captured at 3000 fps.

The dispersion of particles at the air-water interface was similar to that of Máté et al. (1996) who also used micron-sized particles. In a typical experiment the glass container was filled with water to a depth of 20 mm and 3 mL of spreading solvent was poured on the surface of the aqueous phase. Approximately 0.25 g of hydrophobized particles were evenly sprinkled with a spatula onto the spreading solvent, which was left to evaporate for at least 15 min. After complete evaporation of the solvent, the particle monolayer was subjected to compression by movement of the mobile barrier and the surface pressure was recorded as a function of the area per particle in the film. To assess the interaction of a bubble with the interface, a capillary was placed in the subphase at a depth such that a bubble of 2 mm in diameter was just in contact with the interface. The high-speed camera was used to capture the interaction of the bubble with the particle-laden interface. The movement of the particles was tracked during the coalescence of the bubble and during the compression and expansion of the particle layer.

Previous studies have related the surface pressure change in the particle-laden interface to the mean area of each particle in the monolayer (Aveyard et al., 2000). The packing factor was used to describe the coverage of particles on the bubble surface in studies of controlled bubble coalescence (Bournival and Ata, 2010). It is defined as the ratio of the surface area covered by particles to the total surface area (i.e., surface area covered by particles and the voids). To relate the particle packing to the properties of the interface, both the surface pressure and particle packing were measured and analyzed by assessing the top view of the recordings captured by the high-speed camera.

### Analysis of surface pressure isotherms

The surface pressure was measured by a Wilhelmy plate in conjunction with an electronic balance as previously described. The data stream was corrected using cubic spline to remove the noise in the surface pressure measurements. The data was fitted using Spyder (Spyder IDE, version 3.1.4). The software is an open source IDE (integrated development environment) based on Python programming language. The code used is given in the Supporting Information.

### Image analysis and particle tracking

The monolayer and individual particles were imaged using a high-speed camera. Using the recorded video frames as an input the packing factor, defined as the percentage of area covered by particles, was calculated using the ImageJ2 software (NIH). ImageJ2 allowed the frames to be transformed into a binary image by applying a threshold turning particles into black objects and leaving voids as white objects. A summation of these black areas was calculated from each frame and then used to calculate the packing factor by dividing it by the total surface area. Considering the small surface area observed it is important to note that the packing factor may be viewed as a local property rather than the overall packing factor. It was taken in the vicinity of the Wilhelmy plate.

Particle movement across the air-water interface was tracked using Tracker (version 4.97, Open Source Physics Project). Tracker is capable of gathering data from video recordings on the basis of a time and a coordinate system (Brown and Christian, 2011). The program allowed the particles to be tracked both with an autotracker function and a manual tracking function.

Video files that were imported into Tracker required the scale to be calibrated, for which a point of interest within the video with known measurements was identified. For experiments involving the coalescence of a bubble, the capillary tube, which is easily distinguishable and has a known outer diameter of 1.04 mm, was used. Compression and expansion videos were calibrated using the largest particles where the sizes were afterwards crosschecked with other particles. The largest particles were defined as having a size of 92.5 μm (corresponding to the *D*_90_). Occurrence where two of these particles were found adjacent was then defined as twice the *D*_90_. Coordinates were established and defined within the Tracker software to understand how particles moved through the video frames. For tracking the motion of particles following the coalescence of a bubble with the interface, the origin of the coordinate system was defined as the center of the capillary tube. As all compression and expansion videos had the same orientation the origin was defined in all experiments as the top right corner, ensuring continuity during analysis of the different videos. All y-axes were vertically orientated while all x-axes were horizontally orientated when compared with the video orientation.

The primary method used to track particles within Tracker was autotracker due to the quantity of frames requiring analysis. The manual approach was used when the defined automark level was higher than the matching score as calculated by the software. Particles identified for tracking had their center point identified within the software. The autotracker function subsequently defined both a template [characterizing the red, blue, and green (RGB) pixels inside] and a search area within which the particle was searched for in the following frame. The sum of squares of the RGB differences between the template and the search area is inversely proportional to the match score. The speed of the particles was calculated by the software according to the positions of the particles in each frame.

## Results and discussion

### Relationship between the normalized area and the packing factor

**Figure 2:**
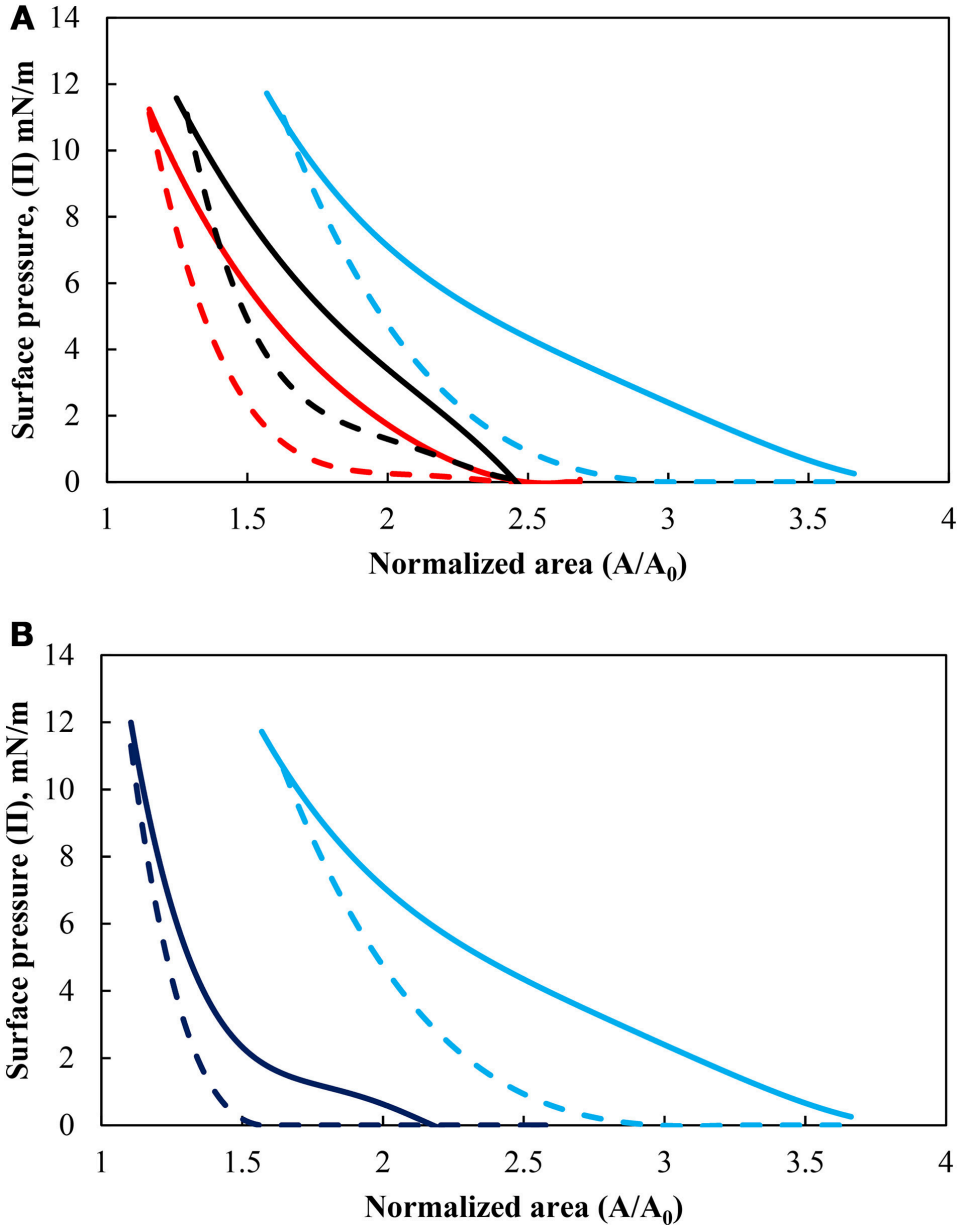
**(A)** Surface pressure isotherms of hydrophobized particles (θ_eq_ = 75°) at pH values of 3 (red), 5.8 (black), and 9 (light blue) and **(B)** surface pressure isotherms for particles hydrophobized with contact angle of 43° (dark blue) and 75° at pH 9 (light blue). Solid lines are the compression cycle while dashed lines correspond to the expansion cycle.

The influence of the pH of the subphase on the surface pressure for the more hydrophobic particles (θ_eq_ = 75°) is shown in Figure 2A while the effect of particle hydrophobicity, at pH 9, is presented in Figure 2B. The solid lines indicate the compression cycles and dashed lines correspond to the subsequent expansion cycles [the second cycle is used since it is more consistent due to the breakdown of long-range and short-range structure (Hórvölgyi et al., 1996)]. The surface pressure is defined as Π = γ_0_ – γ, where γ_0_ is the surface tension of pure water and γ is the surface tension of the interface, representing a difference in interfacial tension. The figures show that the surface pressure increased under compression and decreased under expansion. As the movable barrier compressed the particles into a densely packed film, the surface pressure rose relatively slowly in more dense area but began to rise more steeply with further reduction in the area. The surface pressure eventually reached a maximum value, which ranged from 9.9 to 11.8 mN/m for all conditions studied. It should be noted that the monolayers were not compressed beyond the limiting value as the layers appeared to be unstable beyond the collapse point (i.e., the particles started to detach from the interface). For the three pH values tested, the surface pressure started to increase at a higher normalized surface area upon compression with increases in the pH. A similar behavior was found in the hydrophobicity of the particles with the more hydrophobic particles causing an increase in the surface pressure at a larger normalized surface area as found by others such as Hórvölgyi et al. (1996), Safouane et al. (2007). Changing the pH for the less hydrophobic particles did not have a pronounced effect on the pressure isotherm compared with the more hydrophobic particles. The effect of the change in hydrophobicity is thus discussed only at pH 9.

The packing of particles at an interface can vary widely for any packed layer depending on whether the particles are monodispersed or polydispersed (Bournival and Ata, 2010; Grishaev et al., 2017). As the layer is compressed the structure of the array of particles, assuming they are spherical and monodispersed, should theoretically reach a coverage density of $\sqrt{3}\pi /6$ if surface forces are overcome (further discussed in the following section). As such it is well known that the average separation distance between the particles tends to increase with increases in the pH for glass beyond the isoelectric point (i.e., pH 2–3) (Hórvölgyi et al., 1996; Sastry et al., 1997; Blute et al., 2009). However this concept needs to be discussed in terms of the packing factor and surface forces.

#### Packing of particles at the air-water interface under a compression force

**Figure 3:**
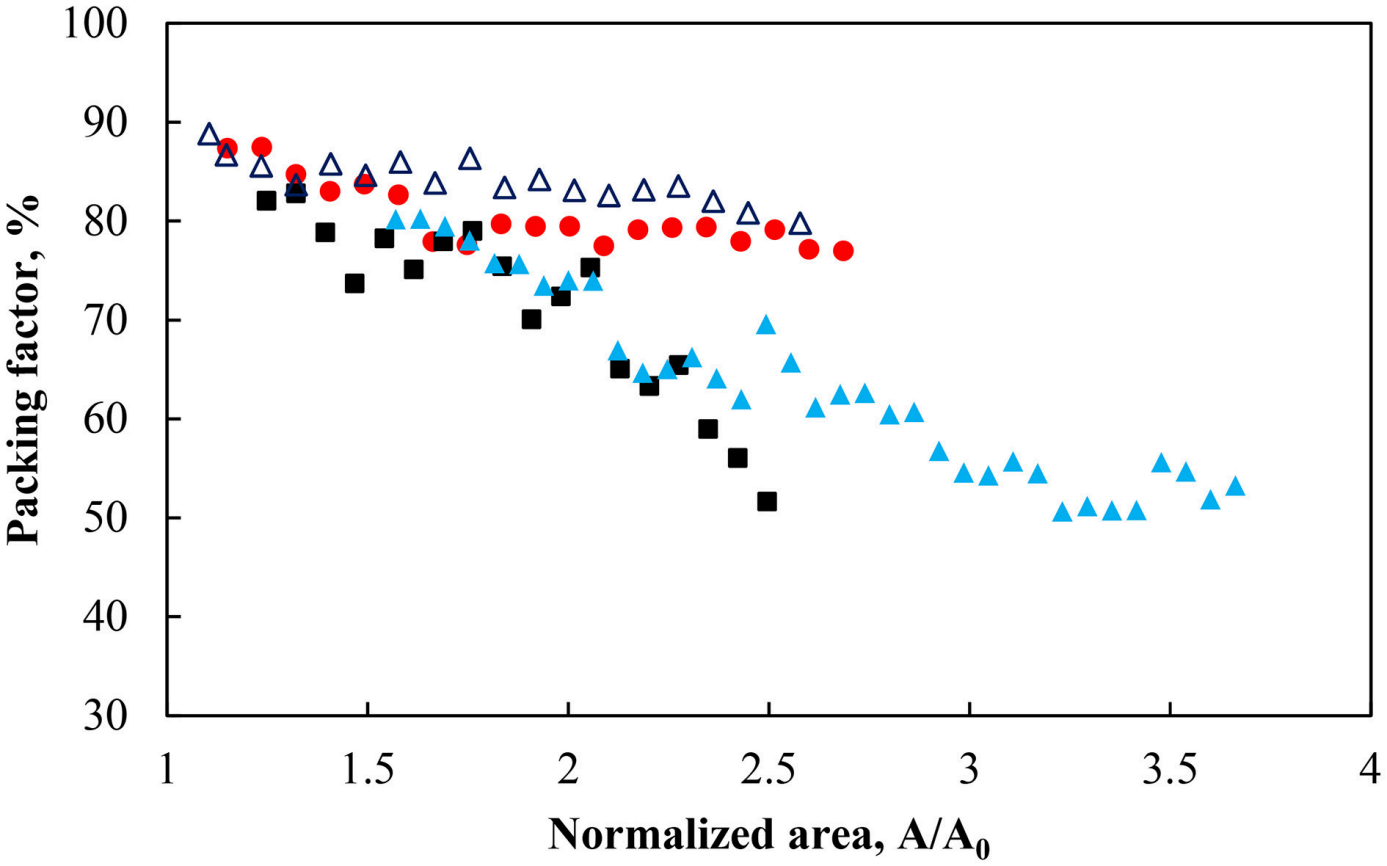
Packing factor during compression for particles hydrophobized with a contact angle of 75° at pH 3 (red solid circle), pH 5.8 (black solid square), pH 9 (light blue solid triangle), and 43° at pH 9 (dark blue triangle).

Figure 2 demonstrated that a different normalized area (i.e., inter-particle average distance) was produced for a similar surface pressure. The average separation distance was then explored in terms of the packing of the particles. Figure 3 shows the packing factor at different pH values during the compression cycles shown in Figure 2. As the surface pressure increased and the average separation distance decreased, the percentage of voids in the particle network decreased resulting in a higher packing factor. Similarly to the work of Huang et al. (2004) with nanoparticles, the micron-sized particles formed rafts at the interface which became more compact and rearranged as the surface pressure increased. As such the average separation distance between the particles was not uniform. However, it provides an average over the entire surface of the trough. On the other hand, the packing factor was a localized measurement. Consequently it can be observed that the more hydrophilic particles and the less densely charged particles (pH 3) produced rafts with more closely packed particles. This discrepancy between the two measurements decreased at higher surface pressures as a result of the gaps between the rafts closing in. It should also be noted that the packing factor was a local packing factor determined in the vicinity of the Wilhelmy plate while the normalized area considered the entire area of the monolayer. Such distinction may explain that the packing factor for particles with a contact angle of 75° at pH 5.8 and 9 are similar. However, the minimum normalized area before the detachment of particles occurred was larger when the pH of the subphase was higher.

#### Particle interaction force

The behavior of the particles under compression and expansion and the packing factors presented in Figures 2, 3 may be explained in terms of the interaction force between the particles. A variety of theoretical models have been developed to calculate the interaction energy between two particles at an interface. In its simplest form the particles may be considered to be fully submerged (Clint and Taylor, 1992) which may lead to a satisfactory correlation of the force with the surface pressure isotherm. However, differences in force resulting from the particle being exposed to a different phase, such as air or oil, can limit such simplification (Kralchevsky et al., 2001; Danov et al., 2004).

The calculation of the interaction forces was made assuming two identical particles partially submerged at the air-water interface. Due to the number of assumptions made and the limited number of forces considered the calculations represent a semi-quantitative assessment of the interaction force between particles. The interaction forces included the van der Waals force (*F*_*vdW*_), the electrostatic force (*F*_*el*_), and the capillary force (*F*_*l*_). The equations for these three forces are given as (Bournival et al., 2016):

(1) $$\begin{array}{r} F_{vdW}=-\frac{A_{eff}r_{p}}{12h^{2}}f\left(p\right) \end{array}$$

(2) $$\begin{array}{r} F_{el}=\frac{6\varepsilon_{air}q_{water}^{2}}{4\pi \varepsilon_{0}\varepsilon_{water}^{2}\kappa^{2}h^{4}} \end{array}$$

(3) $$\begin{array}{r} F_{l}=-2\pi \gamma r_{p}B^{\frac{5}{2}}S^{2}K_{l}\left(\lambda_{c}l\right) \end{array}$$

where *A*_*eff*_ is the effective Hamaker constant, *r*_*p*_ is the radius of the particles, *h* is the separation distance, *f(p)* is a function which corrects the van der Waals force for retardation effects and takes the form:

(4) $$\begin{array}{r} f\left(p\right)=\left(1+3.54p\right)/{\left(1+1.77p\right)}^{2};\;p<1\\ f\left(p\right)=0.98/p-0.434/p^{2}+0.067/p^{3};\;p>1 \end{array}$$

where $p=\frac{2\pi h}{\lambda }$ and λ is the retardation length scale. The effective Hamaker constant was given as

(5) $$\begin{array}{r} A_{eff}=A_{12}+f^{2}\left(3-2f\right)\left(A_{13}-A_{12}\right) \end{array}$$

in which *f* is the linear fractional immersion height of the particles and *A*_12_ and *A*_13_ are the Hamaker constants in the gas and the water phase, respectively. In Equation (2), ε_0_, ε_*air*_, and ε_*water*_ are the permittivity of vacuum, the relative permittivity of air, and of water, respectively. κ is the Debye-Hückel parameter and *q*_*water*_ is the charge of the immersed section of the particles, which follows

(6) $$\begin{array}{r} q_{water}=2\pi r_{p}\kappa^{-1}\sigma \tau_{water}\sin\theta \end{array}$$

where σ is the surface charge density, τ_water_ is the degree of surface group dissociation on the particle and θ is the contact angle of the particles. The zeta potential was used as a substitute for the surface potential in the calculation of the surface charge. The zeta potential were assumed to be −5, −40, and −70 mV at pH 3, 5.8, and 9, respectively (Bergna and Roberts, 2006). The degree of surface group dissociation of a particle in water was assumed to be 0.02 as it is expected to be much lower than 0.53, the maximum value obtained in polysilicic acids (Shchipalov, 1999; Bournival et al., 2016). The capillary force (Equation 3) is a function of γ, the surface tension, *B*, the Bond number, *S*, a sphere constant, and *K*_*l*_, a modified Bessel function, which was approximated 1/(λ_*c*_*l*) $where\;\lambda_{c}=\sqrt{\left(\rho_{l}-\rho_{g}\right)g/\gamma }$ is an inverse capillary number with ρ_*l*_, and ρ_*g*_ representing the densities of the liquid phase and gas, *g* is the gravitional acceleration, and *l* is the separation distance of the particles from the center of mass as previously reported (Chan et al., 1981; Ata, 2008; Bournival et al., 2016).

**Figure 4:**
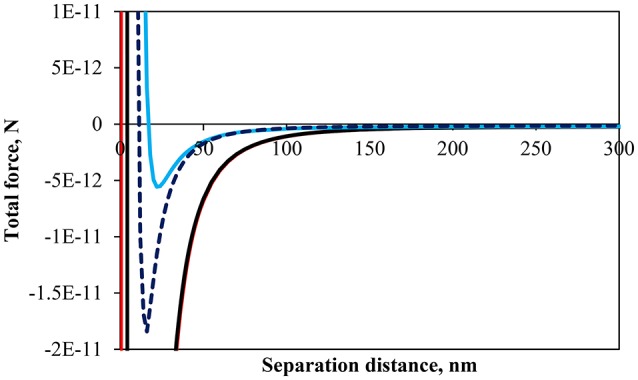
Calculated total interaction force between two identical particles with a contact angle of 75° at an air-water interface at pH 3 (red), pH 5.8 (black), pH 9 (light blue), and particles with a contact angle of 43° at pH 9 (dark blue dashed line).

Figure 4 shows the total interaction force, which is a summation of Equations (1)–(3), for particles of 64 μm in size and a contact angle of 75° at pH 3, 5.8 and 9 as well as a contact angle of 43° at pH 9. In all cases the interaction force appears to be slightly negative at large separation distances (see Figure 4 for separation distances >150 nm). An individual evaluation of the three forces shows that the capillary pressure is acting at larger separation distances due to the strong effect of gravity on these large particles. At a closer separation distance the particles are, theoretically, increasingly attractive as the pH decreases. The calculations are consistent with the fact that the isoelectric point for glass lies approximately between 2 and 3, which, implies that the repulsive force should become increasingly important as the pH increased due to increases in the density of negative surface charges (Behrens and Grier, 2001). It should be noted that the zeta-potential used in the calculation of the repulsive force was assumed to be the same as non-esterified glass beads as in the work of Hórvölgyi et al. (1991) and Laskowski and Kitchener (1969) for methylated silica. This assumption stems from the fact that the number of silanol groups on a silica surface is in the order of 5.2 per nm^2^ (Zhuravlev, 2006). The esterification of silica produces a coating with an average density of 1.57 ester groups per nm^2^ (Ossenkamp et al., 2001). Since the ester is formed through the reaction of a silanol group it was expected that the number of silanol groups would be well above 0.5 per nm^2^, which is the limit under which electrophoretic mobility of particles is affected according to Blake and Ralston (1985). The calculations of the interaction force, although semi-quantitative, corroborate the results shown in Figures 2A, 3 where the surface pressure and the packing factor differed for the different pH at any given normalized surface area. Noteworthy is the fact that the minimum normalized area, in Figure 3, before the removing of particles from the interface decreased with the pH. It indicates that the detachment of particles occurred when particles were more loosely arrayed and the total force was less attractive.

Figure 4 also includes the calculation for a less hydrophobic particle (θ = 43°) at pH 9. The interaction force curves are relatively close to one another although the difference in fractional immersion would slightly affect all forces indirectly (cf. *f, q*, and *S*). However, it should be noted previous studies have shown that a reduction in surface wettability increased the repulsive force between particles (Hórvölgyi et al., 1991; Horozov et al., 2005; Safouane et al., 2007; Blute et al., 2009), which is consistent with the results of Figure 2B. Although the portion of the particle immersed in water is larger for the less hydrophobic particles the charge of the water immersed section is larger for the more hydrophobic particles due to the screening of charge for κ*a* >>1 (Aveyard et al., 2002). Moreover, the length of the alkyl coating (similar to flocculation by neutral polymers) (van de Ven, 1989), which imparts the hydrophobicity to the particles, and hydrophobic forces (e.g., Wang and Yoon, 2004) were not taken into account in the calculations and could explain the similar pressure isotherms between the less hydrophobic particles and the more hydrophobic particles at pH 3, unlike the calculated interaction forces.

Furthermore, a model may be fitted to correlate the packing factor with the surface pressure (Horvölgyi et al., 1999). The expression, which was developed by Fainerman et al. (2006), applies to both molecules and particle monolayers as follows:

(7) $$\begin{array}{r} \Pi =-\frac{kT}{\omega_{0}}\left[\ln\left(1-\frac{\omega }{A}\right)+\left(\frac{\omega }{A}\right)\right]-\Pi_{coh} \end{array}$$

where Π is the surface pressure, *k* is the Boltzmann constant, *T* is the temperature, ω_0_ is the molecular area of a solvent molecule (taken as 0.18 nm^2^ for water Fainerman et al., 2006), ω*/A* is the packing factor in which ω is the average area of a particle, *A* is the surface area occupied by each particle, and Π_*coh*_ is the cohesion pressure (i.e., the pressure arising from the interaction of the components of the monolayer). The cohesion pressure may be expressed as *kT*/ω_0_ ln $f_{0}^{H}$ with $f_{0}^{H}$ being the activity coefficient. The cohesion pressure can be estimated from the approximation given by Fainerman et al. (2003) as $f_{0}^{H}$ = *a*(ω/*A*)^2^ in which *a* is the Frumkin interaction parameter for non-ideality. However, to simplify the calculations the cohesion pressure was assumed to be constant (Fainerman and Vollhardt, 1999) and fitted for each system, i.e., a constant affected by the hydrophobicity of the particles and the pH of the subphase.

**Figure 5:**
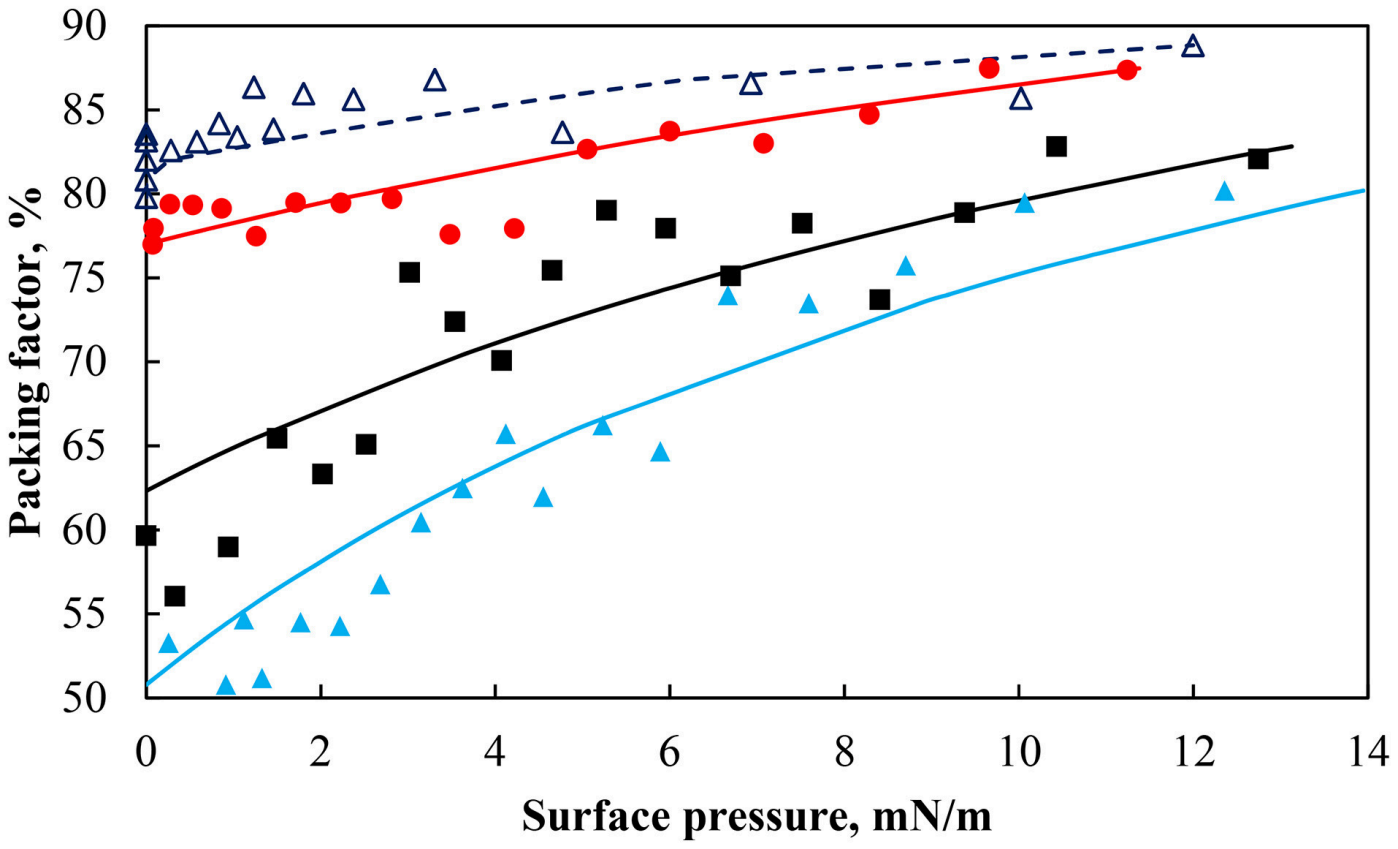
Particle packing factor as a function of surface pressure for particles of contact angle 75° at pH 3 (red solid circle), pH 5.8 (black solid square), pH 9 (light blue solid triangle), and a contact angle of 43° at pH 9 (dark blue triangle). Lines are lines of best fit of Equation (7).

The packing factor as a function of the surface pressure is shown in Figure 5 for the hydrophobic (θ = 75°) and less hydrophobic (θ = 43°) particles at different pH along with the fitted model. As expected an increase in the surface pressure produced an increase in the packing factor albeit at a different rate. For the same change in the surface pressure, the packing factor at pH 9 increased approximately from 50% to more than 75%, while at pH 3 the overall increase in the packing density was around 15%. The smallest change in the packing factor was observed for the less hydrophobic particles at pH 9. It appears that the different systems had different total pressure (Π+Π_*coh*_). As seen in Table 1 the values of the cohesion pressure increased with the decrease in the pH value and would explain the lower packing factors for particles in more acidic suspensions and for particles of lower hydrophobicity.

**Table 1:** Cohesion pressure for the packing of glass particles of different hydrophobicity partially submerged in water at different pH.

**Particle contact angle (°)**	**Subphase pH**	**Cohesion pressure (*Π_*coh*_*) (mN/m)**
75	3	15.6 (0.3)
75	5.8	8.0 (0.5)
75	9	4.7 (0.4)
43	9	22.9 (0.7)

*The standard error of the estimate on the cohesion pressure is in brackets*.

The cohesion pressure is related to the interaction energy (or force) between the particles (Hórvölgyi et al., 1994) and should be consistent with the calculations of Figure 4 in terms of the minimum force. The cohesion pressures seem in agreement with the interaction force curves since the more attractive the interaction force, the larger is the cohesion pressure. The effect of particle hydrophobicity on the cohesion pressure has been studied by Fainerman et al. (2006) who found that less hydrophobic particles had a higher cohesion pressure, which is consistent with the result presented in Table 1. However, the difference was not as marked in the surface force calculations. It should be noted that the calculations did not take into account the hydrophobization method, which may have affected the actual interaction forces of the particles.

### Particle movement during compression

#### Particle movement under different compressing speeds

Video recordings of the particle layer in the vicinity of the Wilhelmy plate were taken to study the re-organization of particles under different compression speeds. Particles were seen to move across the field of view of the camera as they moved toward the Wilhelmy plate.

**Figure 6:**
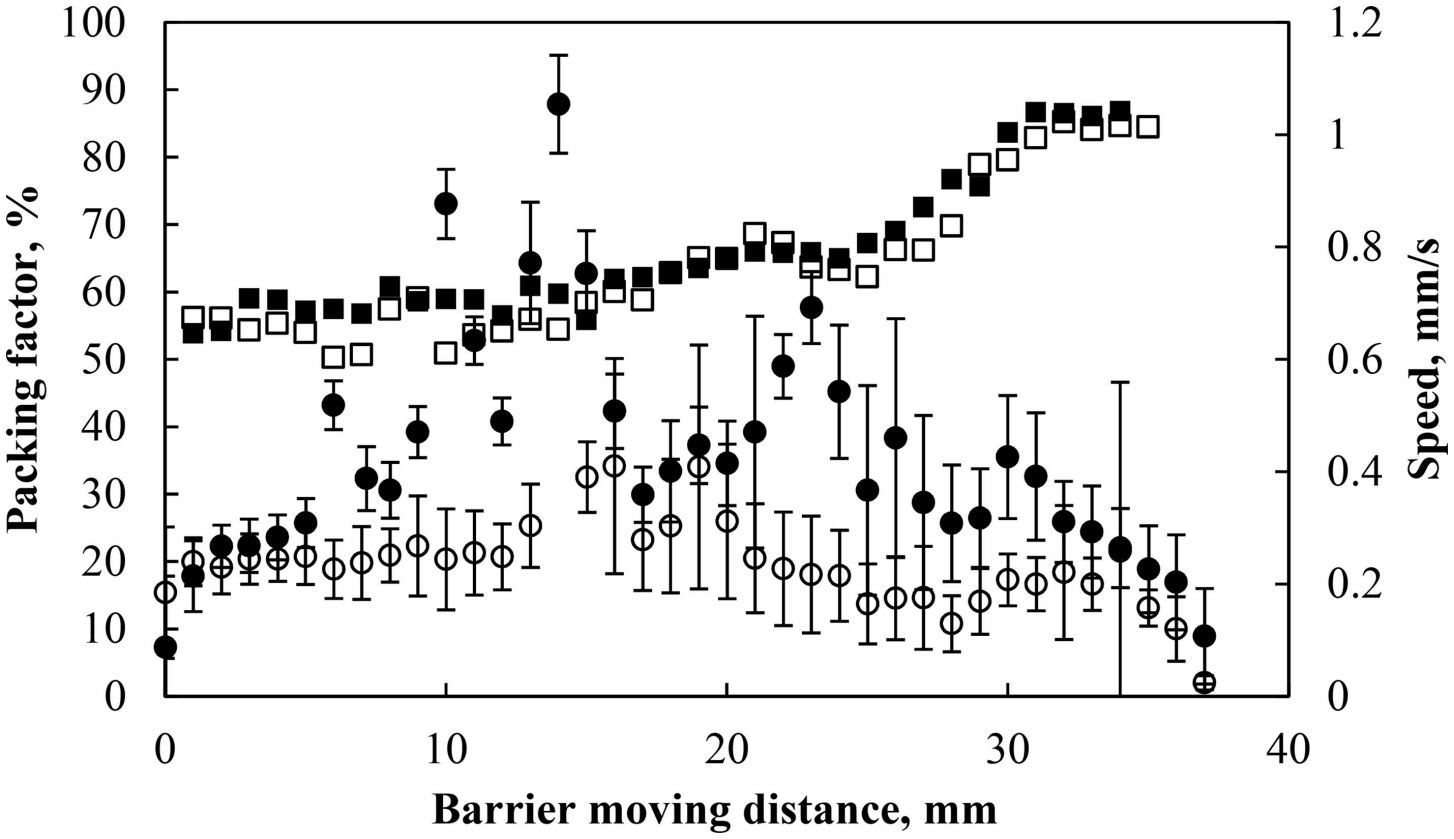
Packing factor (black solid square) and particle speed during compression (black solid circle) of particles hydrophobized at 75° and pH 5.8 with a barrier speed of 0.8 mm/s and packing factor (black square) and particle speed (black circle) under the same conditions with a barrier speed of 0.45 mm/s. The particle speed was averaged over 5 particles and the error bars represent one standard deviation of the averaged 54 and 40 data points at 0.8 (black solid circle) and 0.45 (black circle) mm/s, respectively.

Figure 6 shows the particle speed and the packing factor as a function of the barrier displacement from its initial position at two different barrier compressing speeds: 0.45 and 0.8 mm/s. The packing factor started at ~55% and reached 85% after a 35 mm displacement of the barrier irrespective of the barrier speed. The average speed of particles (circles in Figure 6) was averaged over 5 particles. In each case the particles momentarily reached a speed that was close to the barrier speed but much of the movement of the particles (around the Wilhelmy plate) was much slower due to the restriction of other particles (Jeng et al., 2002). The speed of the particles was further reduced as the packing factor reached its maximum value, which corresponded to the removal of particles from the interface. In general the particles traveled at speeds below the compression speed and the traveling speed decreased as the particles get close packed.

#### The effect of particle shape and size on particle displacement during compression

**Figure 7:**
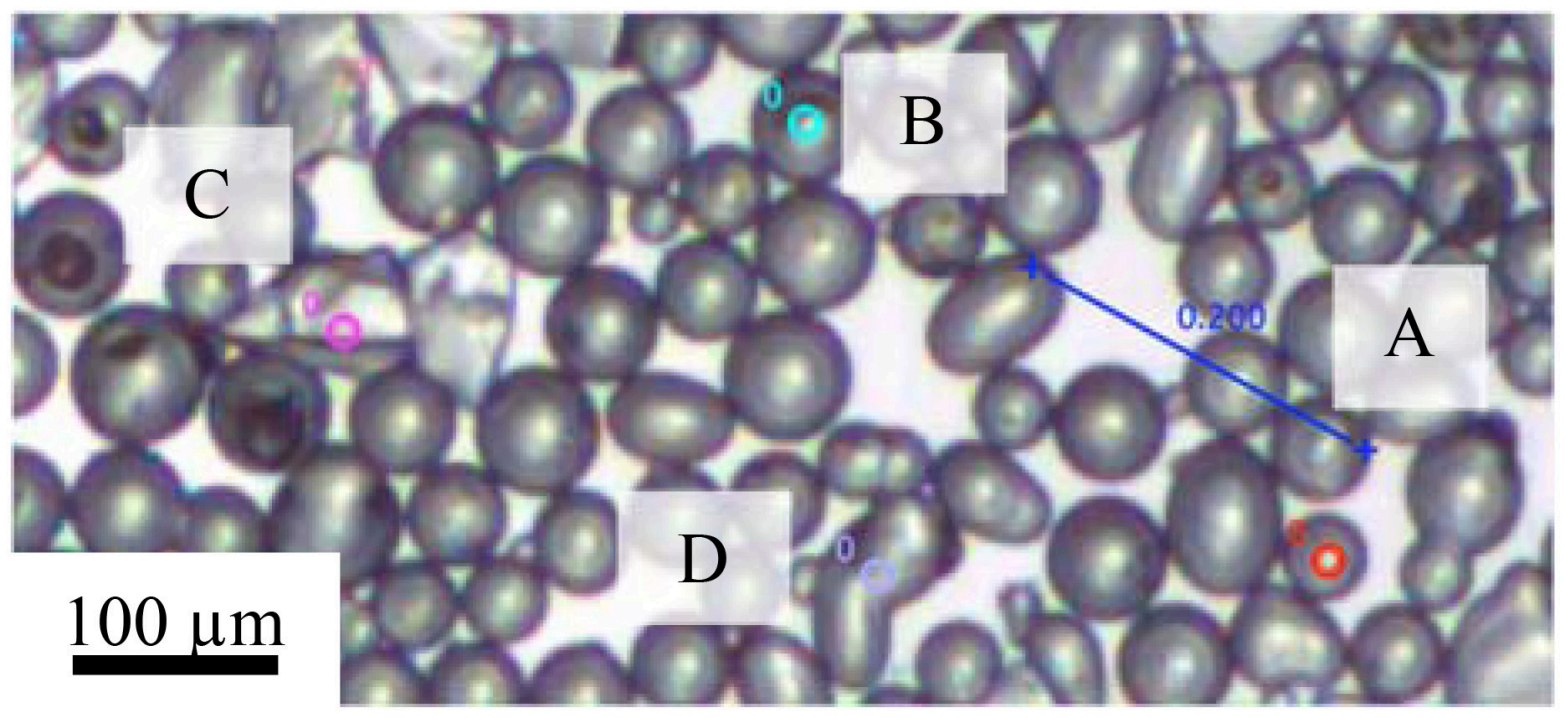
Selected particles in the calculation of correlation between particle speed with size and shape: **(A)** (*d*_*e*_ = 44.9 μm, *r* = 1); **(B)** (*d*_*e*_ = 60.4 μm, *r* = 1); **(C)** (*d*_*e*_ = 81.0 μm, *r* = 0.6), and **(D)** (*d*_*e*_ = 80.7 μm, *r* = 0.6). The measurements were conducted on particles with a contact angle of 75° and at pH of 3.

After the initial spreading of the particles and the evaporation of the spreading solvent, small rafts of particles with high stability moved uniformly. During compression, the small rafts came into contact with each other. For particles at a low pH the repulsive force between particles was weaker and the small rafts, under the force supplied by the barrier, rearranged to form a single raft (discussed in section The Motion of Particles Raft During Compression). Figure 7 shows a frame from a video on the compression of the more hydrophobic particles at pH 3. Particles of different shapes were selected to study the effect of particle shape in the motion of particles with a monolayer. The particles were characterized by their equivalent diameter (*d*_*e*_):

(8) $$\begin{array}{r} d_{e}=\sqrt{\frac{particle\;area}{\pi }} \end{array}$$

and their aspect ratio (*r*) (Barreiros et al., 1996):

(9) $$\begin{array}{r} r=\frac{minimum\;Feret\;diameter}{maximum\;Feret\;diameter} \end{array}$$

The particles did not move independently but instead moved coherently with the surrounding particles forming the raft. As a whole the particle layer behaved uniformly during the compression. The correlations between the speeds of four particles found within the same video frame over a period of 15 s were relatively high. During that time period, the local packing factor increased from 77 to 79%.

A correlation factor was used to quantify the strength of the association between the speeds of each pair of particles. The correlation was calculated using the Pearson product-moment correlation coefficient (*c*) between the speed of one particle (*v*_1_) and that of another (*v*_2_) through:

(10) $$\begin{array}{r} c=\frac{\sum \left(v_{1}-\bar{v_{1}}\right)\left(v_{2}-\bar{v_{2}}\right)}{\sqrt{\sum {\left(v_{1}-\bar{v_{1}}\right)}^{2}\sum {\left(v_{2}-\bar{v_{2}}\right)}^{2}}} \end{array}$$

where $\bar{v_{1}}$ and $\bar{v_{2}}$ are averages of *v*_1_ and *v*_2_ respectively. The correlation was higher than 0.98 in all cases, which indicates a strong correlation between speeds of the different particles (value close to 1) and that they are moving in the same direction (positive correlation coefficient). Differences in the shape and size of particles did not result in different behavior of particles at the air-water interface during compression under the test conditions.

#### The motion of particles raft during compression

In addition to examining the compression and expansion behavior of particle monolayers in relation to pH, it is also of great interest to understand the motion of individual particles in the monolayer undergoing compression. The correlation coefficient was used during analysis of compression experiments to measure how particles moved in relation to one another, since they could rotate or fill voids as discussed later.

**Figure 8:**
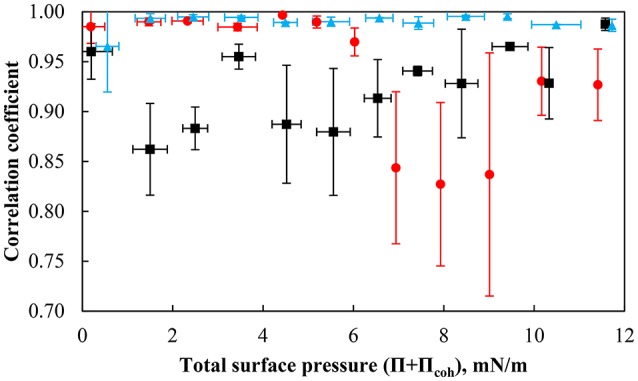
Correlation coefficient of the particles with a contact angle of 75° at pH 3 (red solid circle), pH 5.8 (black solid square), and pH 9 (light blue solid triangle) as a function of the total surface pressure. The error bars represent one standard deviation obtained from evaluating the correlation coefficient between 10 particle pairs.

The average correlation coefficient during compression at different pH as a function of total surface pressure is shown in Figure 8. At pH 9, the correlation coefficient is high around 1, suggesting that the particles move at the same speed and in the same direction. The surface forces exerted between the particles seem to prevent the particles from undergoing substantial rearrangement within the monolayer. The correlation coefficients of the differences in relative speed of two particles obtained at pH 5.8 and 3 at various total surface pressures are quite scattered. The data shows that the difference in the relative speed of the particles moving through the layer is largest in the less dense particle layer for a total surface pressure ranging between ~2–6 mN/m corresponding to a packing factor of 50–70% while more variation at a total surface pressure >6 mN/m was found at pH 3 for a packing factor of ~85–88%. It is worthwhile to mention that the monolayer rearrangement at pH 3 could only be activated under a sufficient compressive loading. The ease at which particle rearrangement takes place follows the order of the interaction force with the conditions creating the less repulsive environment being more conducive to the rearrangement of particles at higher packing factors (i.e., smaller average separation distances) and may be related to the concept a yield stress resulting in building a monolayer network as indicated by the pressure isotherms and the interaction force. Finally, although particle rearrangement occurs, the correlation coefficient is still relatively high, which is consistent with the particles moving nearly uniformly at the interface.

**Figure 9:**
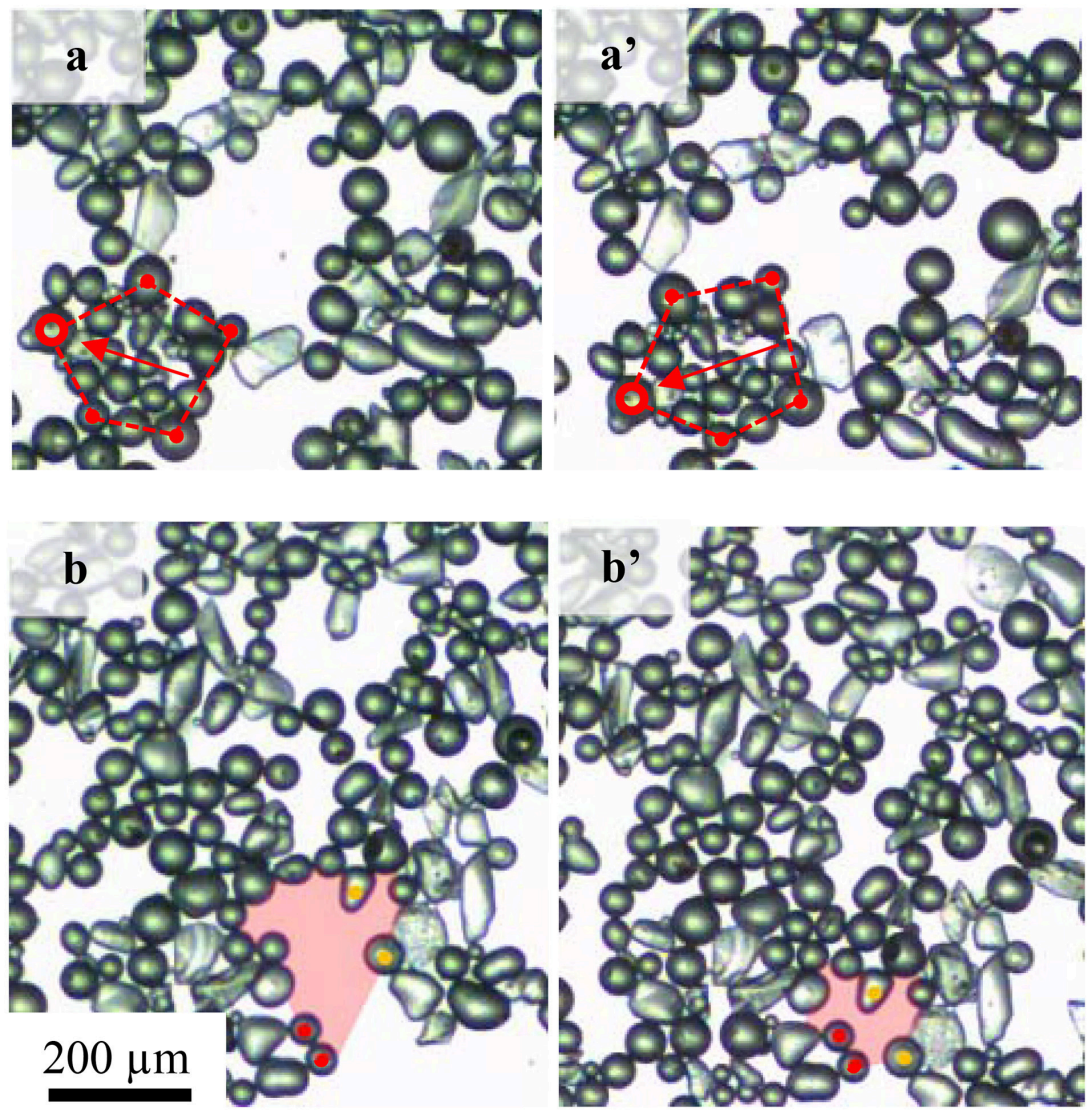
Two particle arrangements causing the correlation coefficient drop: **(a,a')** rotation of a group of particles and **(b,b')** void collapse. Videos are available as supporting document.

Two distinct particle behaviors were observed to cause a reduction of the correlation coefficient. Figure 9 shows the particle rearrangement under compression at the natural pH (pH 5.8) for particles with a contact angle of 75°. As the layer is compressed, individual particle rafts approached each other to form larger agglomerates. In some cases, (Figure 9a), individual clusters were observed to rotate as a whole in such a way that they fitted into the big interstitial voids between the particles. This phenomenon was commonly observed in the layers with lower packing densities where small particle rafts float relatively freely at the air-water interface. A drop in the correlation coefficient here is caused by a difference in the direction in which the particles travel. In contrast, at relatively higher packing factors, voids collapsed as highlighted by the change in the shaded area for Figure 9b. The change in the correlation coefficient is the result of variations in the traveling speed between particles as illustrated in Figure 9b where particles marked in red remained almost stationary and particles marked in orange traveled as a result of the void collapse. Rotation of particle groups caused the correlation to drop more than the collapse of bare areas.

### Interaction between a bubble and particle-laden interface

#### Coalescence of bubbles with a particle-laden interface

Several investigations (Ata, 2009; Bournival et al., 2016) have studied experimentally the effect of the physicochemical properties of particles on the stability and coalescence behavior of air bubbles in water. While in the mentioned studies the bubble coating process reflects real conditions, it was not possible to manipulate the packing of the particles on the surface. The present experimental setup allows fine control of particle monolayer formation at the air-water interface, making it possible to study the role of surface pressure and the packing factor in bubble coalescence and the behavior of the interface after the rupture coalescence.

**Figure 10:**
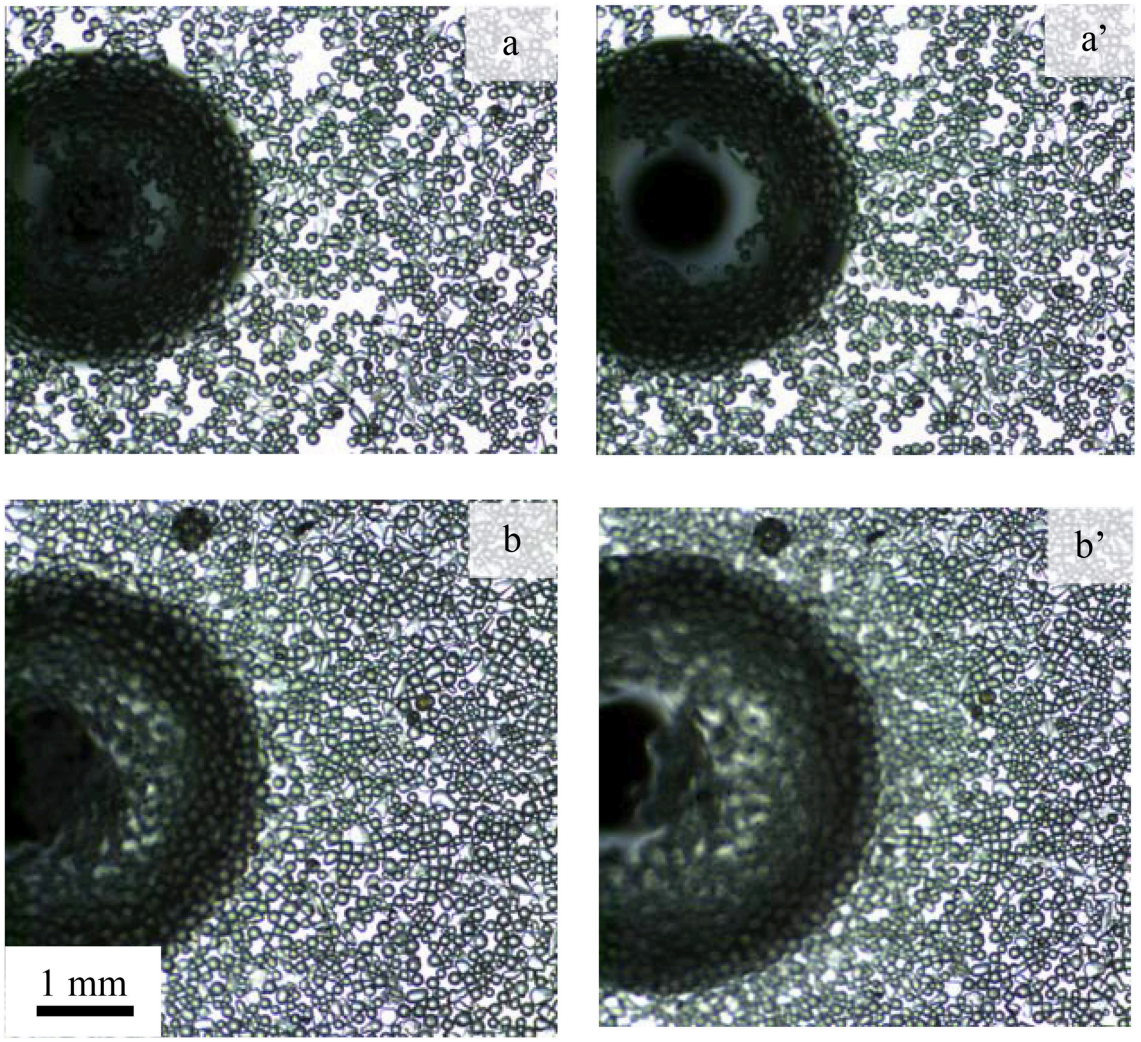
Particle layer under the influence of a bubble forming a bare contact region. Photographs show **(a,b)** the contact of the bubble with the interface and **(a',b')** the interface prior to coalescence for initial packing factor of **(a,a')** 69% and **(b,b')** 81% in an experiment using hydrophobic particles (contact angle of 75°) at natural pH.

Figure 10 shows the interaction of a single bubble with the air-water interface sparsely (a,a') and heavily (b,b') laden with particles of contact angle 75° at natural pH. In Figure 10 the left (a,b) and right (a',b') images present the bubble just reaching the interface and before coalescence, respectively. Both layers contained the same number of particles, but compressed to different surface pressures, 2.1 and 6 mN/m corresponding to packing factors of 69 and 81%, respectively. An air bubble was introduced beneath the layer and allowed to grow slowly. The bubble approached the interface and started to push the surface upward due to the buoyancy force of the bubble and the gradual increase in the bubble volume. The deformation of the interface led to the formation of a bare region at the top with the displaced particles thickly accumulating at the lower part of the convex interface. Eventually, the film ruptured due to the local particle packing defect (Pugh, 1996; Wasan et al., 2005; Planchette et al., 2013; Bournival et al., 2016). It appears that the initial packing factor significantly affected the formation of the bare region. Compared to the more densely packed interface, the bubble easily pushed the particles from the contact region forming a larger void in the low density film. For lower particle packing densities the lateral mobility of particles was much greater than that of higher density particle packing, which might have allowed the formation of a larger particle-barren area.

**Figure 11:**
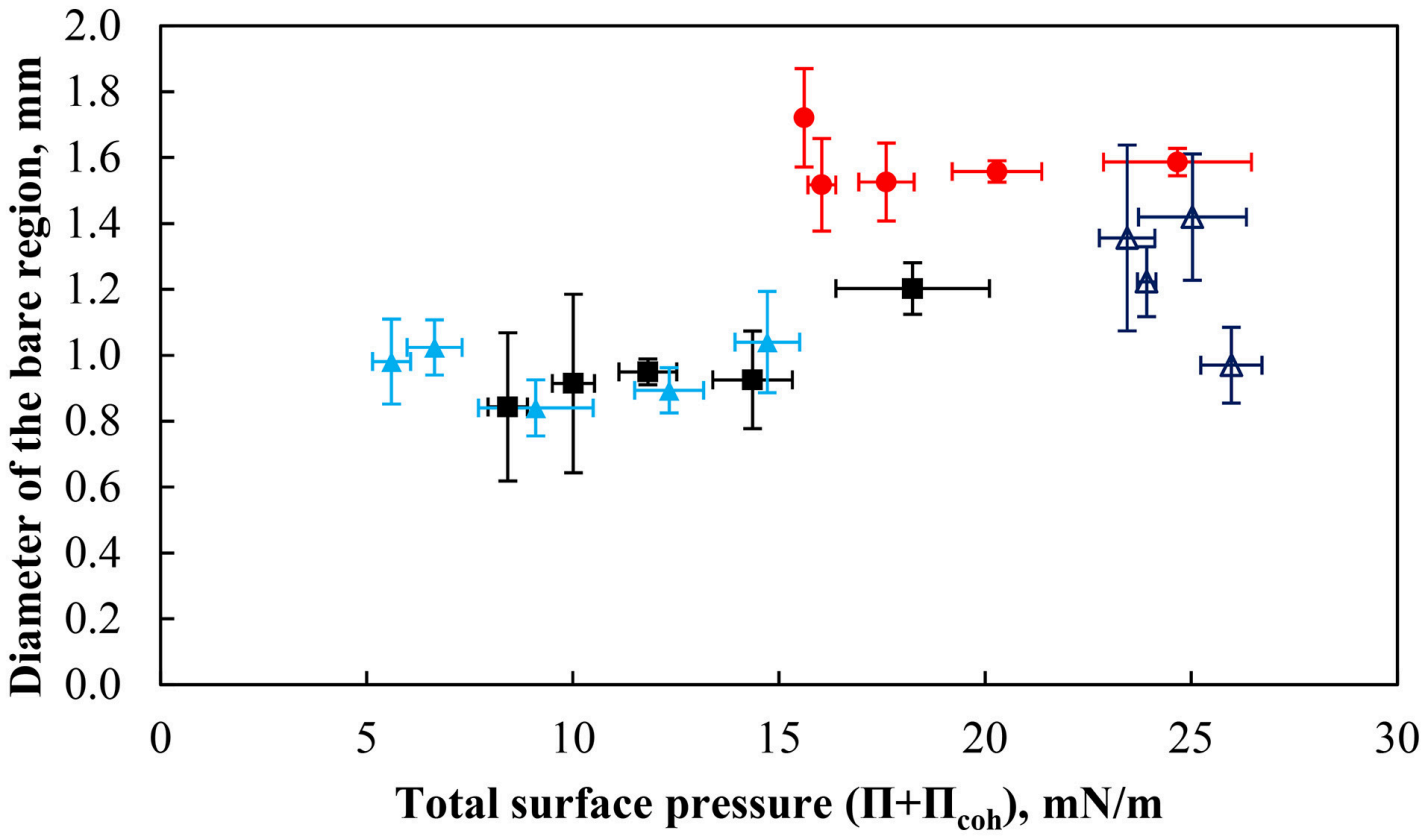
The equivalent diameter of bare contact region prior to coalescence for bubbles of 2.00 ± 0.12 mm under the interface as a function of the total surface pressure of particles with a contact angle of 75° at pH 3 (red solid circle), pH 5.8 (black solid square), and pH 9 (light blue solid triangle) as well as particles with a contact angle of 43° and at pH 9 (dark blue triangle). The error bars show the standard deviation calculated from at least 6 measurements, which were performed under similar conditions.

Figure 11 shows the equivalent diameter of the bare region formed at the interface just before coalescence as a function of the total surface pressure for strongly hydrophobic particles (θ_eq_ = 75°) at various pH values and the less hydrophobic particles at pH 9. Measurements were repeated a number of times under nearly similar conditions and averaged. As mentioned previously, the particles were densely packed at the interface at pH 3, and therefore measurements could only have been performed at high surface coverages. As seen in the figure, there appears to be a low variation in the size of the particle-free region for a total surface pressure below ~12 mN/m. Above a total surface pressure of 12 mN/m, which corresponds to a packing factor of ~80%, the size of the defect is dependent on the hydrophobicity of the particles and the pH of the subphase with a low pH value necessitating a larger bare region for coalescence. Thus at higher surface coverages, a higher capillary pressure, as presented by Kaptay (2006), is needed for coalescence to occur. Since the bubble diameter is constant (i.e., the capillary pressure is not changed), a greater defect is needed for coalescence, which resulted in longer bubble coalescence times.

The formation of a defect is important to initiate bubble coalescence. As the bubble approached the interface, a thin liquid film between the bubble and the free surface must have developed (e.g., Wasan et al., 1992). The liquid in the film drained out gradually and the film ruptured once it reached a critical thickness. In the presence of particles, a three phase contact line between the bubble and particle may be established leading to particles forming a bridge in the thin liquid film (Kaptay, 2004; Morris et al., 2011), which may be influenced by the hydrophobicity of the particles and the pH of the liquid phase. The bridging of the particles may result in a significant delay in bubble coalescence time. The effect of particle bridging on bubble stabilization is well established (Dippenaar, 1982). Moreover, particles in the periphery of the bare region can influence the drainage of liquid and particles (Cain and Lee, 1985; Baets and Stein, 1993).

**Figure 12:**
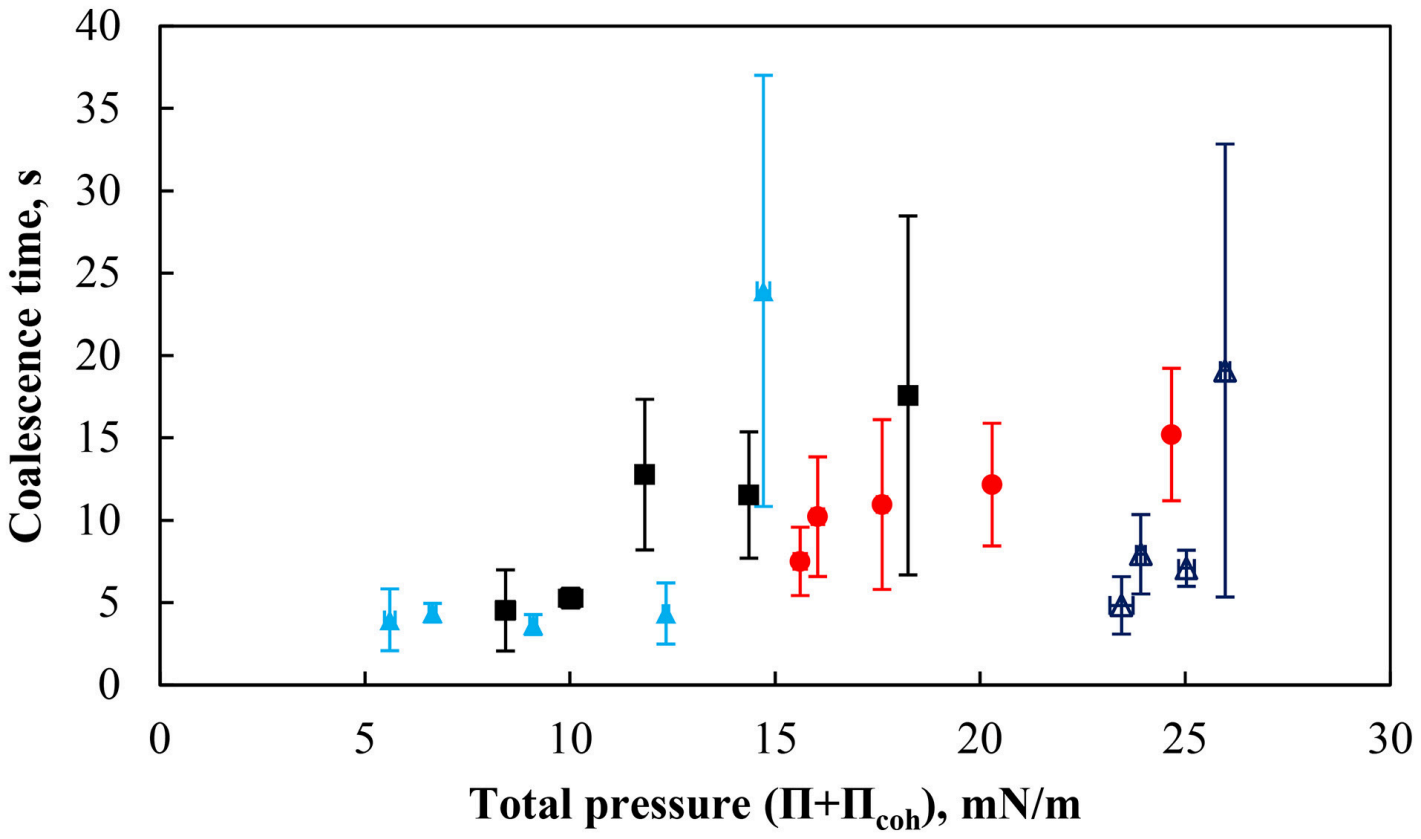
Coalescence time for 2.00 ±0.12 mm bubble as a function of particle packing factor for more hydrophobic particles (75°) at pH 3 (red solid circle), pH 5.8 (black solid square), pH 9 (light blue solid triangle), and weakly hydrophobic particles (43°) at pH 9 (dark blue triangle). The error bars show standard deviation of 6 measurements, which were performed under similar conditions.

Figure 12 shows the coalescence time as a function of total surface pressure for an air bubble beneath the particle monolayer compressed at various packing factors. The coalescence time was defined as the time measured from the first contact of the bubble with the interface and the rupture of the thin liquid film. The coalescence time is generally small at low total surface pressures with times below 5 s. However as the total surface pressure increased above ~12 mN/m the coalescence times started to vary. There was no clear correlation between the coalescence time and the total surface pressure of the combined systems. However, the total pressure within a given system (i.e., pH of the subphase and the contact angle of the particles) affected the coalescence time.

#### Wave propagation and layer healing

**Figure 13:**
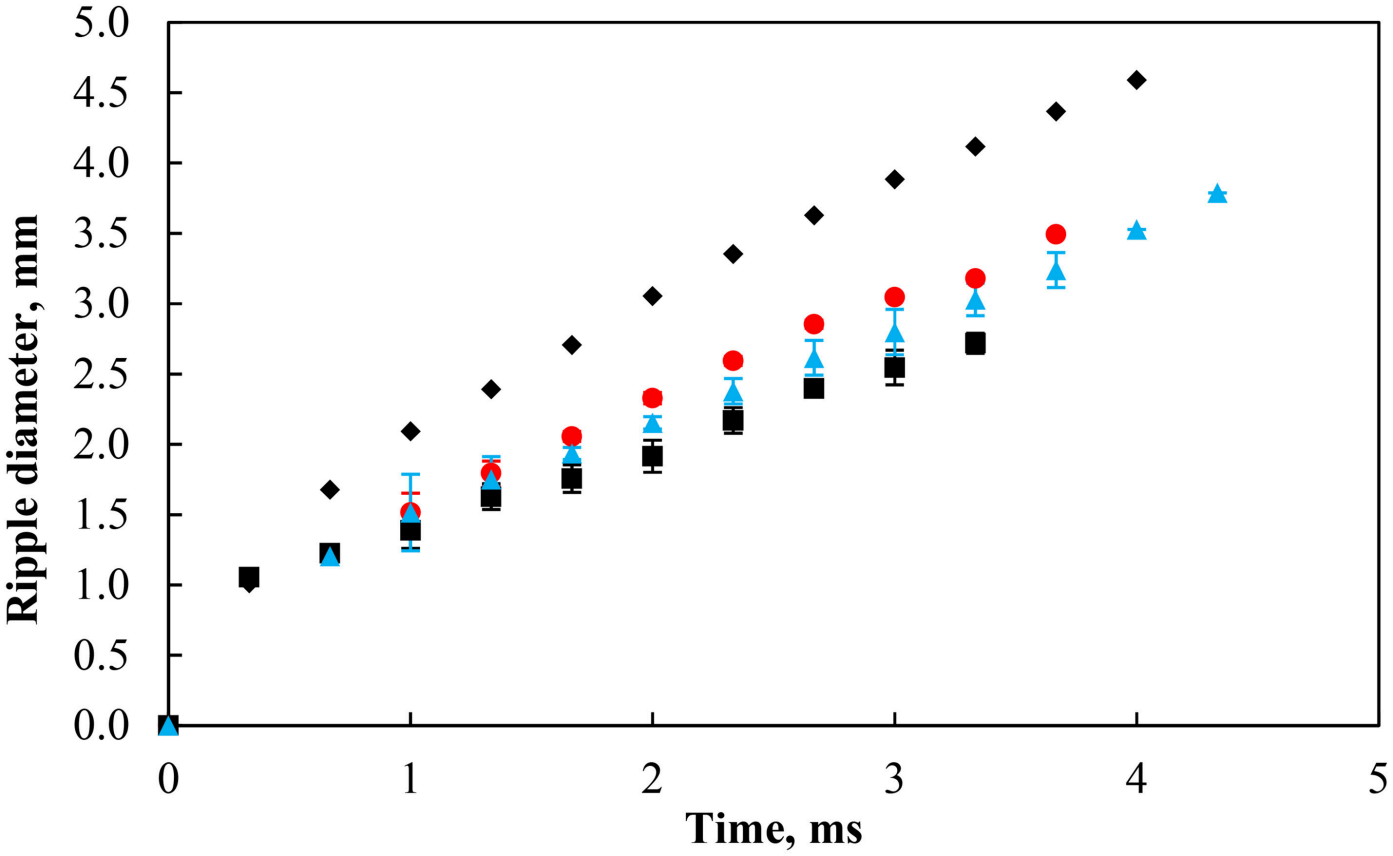
Ripple diameter in pure water (black solid diamond) and with particles at contact angle of 75° with a similar packing factor at pH 3 (red solid circle, 77%), 5.8 (black solid square, 79%), and 9 (blue solid triangle, 79%). The bubble size was 2.00 ±0.12 mm.

Upon the rupture of the thin film separating the bubble and the particle-laden interface, a ripple spreads out from the contact region of the bubble and the interface during which the particles undergo significant rearrangement at the interface. Figure 13 shows the evolution of the ripple diameter with time for a bare air-water interface and a particle-laden interface with similar packing factors produced by the coalescence of a 2 mm bubble. It is clear from the figure that compared to water, the speed of the ripple was considerably reduced by the presence of particles, indicating that particles play an important role in the control of the response of particle-laden interfaces against surface deformations. The figure also suggests that the subphase pH may have a negligible effect on the wave propagation due to slight variations in the initial packing factor and in the size of the bubble. It is yet to be investigated how the size of the bare region (i.e., the number of particles displaced) affects the propagation of the ripple.

**Figure 14:**
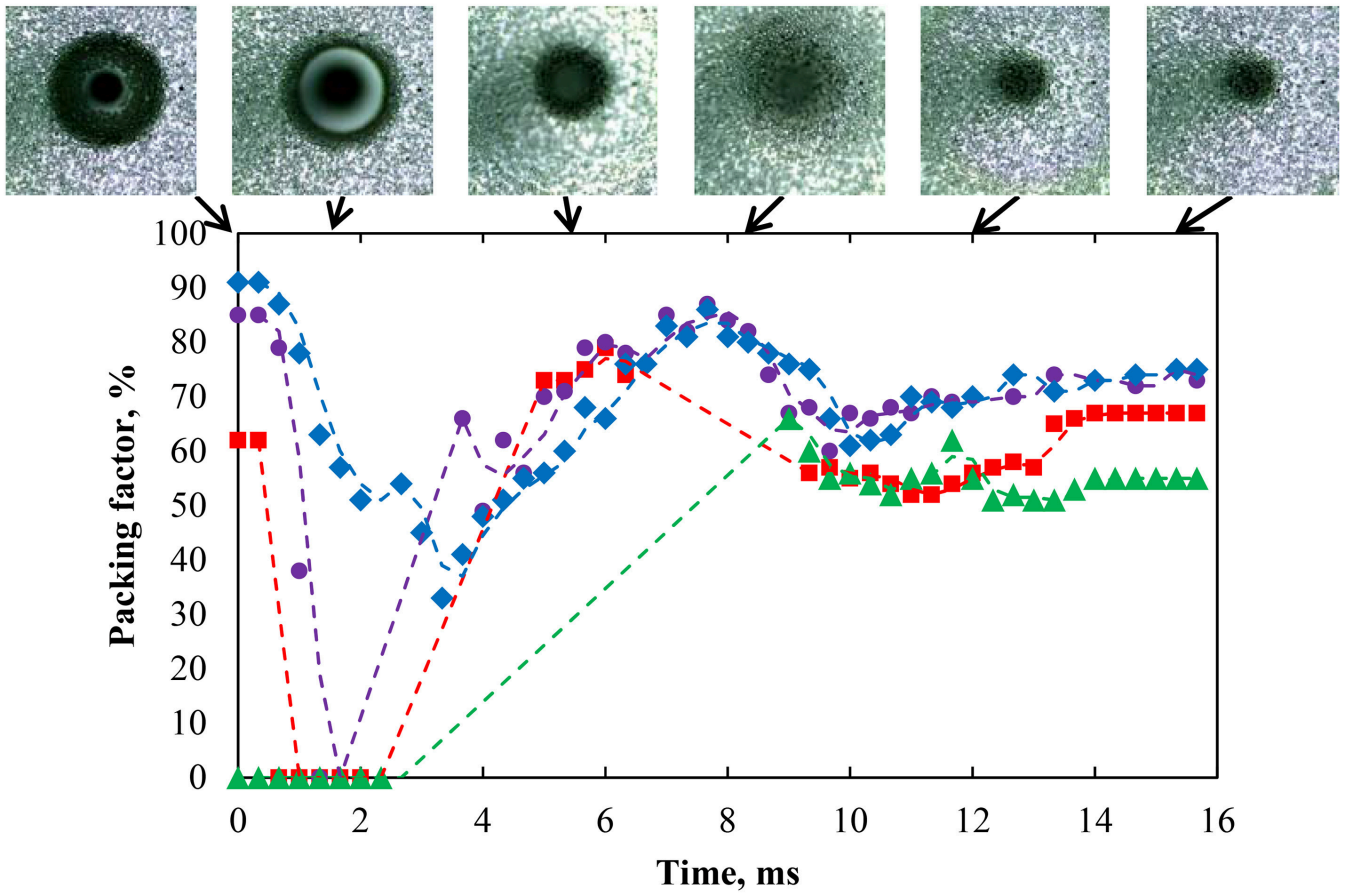
Packing factor of four regions from innermost to outermost—region 1 (green solid triangle), region 2 (red solid square), region 3 (purple solid circle), and region 4 (blue solid diamond)—after coalescence of a bubble with an interface laden with hydrophobized particles (75°) with a packing factor of 82% at natural pH. Region 1 has a radius of 0.76 mm corresponding to most of the bare region while the other regions were at constant surface area. The coalescence time was 2.54 s.

Figure 14 shows the packing factor variation for strongly hydrophobic particles (75°) at natural pH after coalescence. The region of interest was divided into four sections numbered 1–4, starting at the center of the contact area of the bubble with the interface. The width of each section was set to 0.7 mm (note the capillary appears smaller in Figure 14 due to the deformation of the interface by the bubble). The results indirectly quantified the movement of the particles at the interface and the healing of the particle-free region. After the bubble coalesced, the particle packing in region 2 immediately decreased followed by regions 3 and 4. The videos also revealed that particles from regions 3 and 4 moved inward because of the surface pressure difference. According to Figure 5, the region with a high packing factor has a higher local surface pressure, which results in a surface pressure gradient. This surface pressure gradient can cause particles to spread toward the low surface pressure bare region (La Mer and Robbins, 1958; Robbins and La Mer, 1960). The coalescence caused the waves to spread out from the contact region providing kinematic energy to the particles. After the reduction in packing factor, particles from a more distant region moved back to the inner region as a result of wave propagation (Denissenko et al., 2006). As a result, region 2, which was close to the bare region, had a net decrease in the amount of particles while region 1 had a net gain of particles over the timeframe of 15 ms as indicated by the packing factor. The energy of the wave further away from the capillary was reduced because of the viscoelastic property of the interface (Vignati et al., 2003; Cichocki et al., 2004). As a result, the change in the packing factor in regions 2–4 gradually diminished.

## Conclusions

A series of experiments were conducted using a modified Langmuir-Blodgett trough which had a transparent deep glass cell beneath the interface. The aim was to explore the use of such a system to study the interaction of an air bubble with a particle-laden interface, which characteristics could be controlled. The technique enabled the interaction between a particle stabilized air bubble (generated through a capillary in the aqueous phase below the Langmuir-Blodgett thin film) and Langmuir-Blodgett film coated with particles to be studied using a high-speed video together with image analysis. Spherical glass particles (92 μm) with different degrees of hydrophobicity (contact angle with water 75 and 43°) were used in the experiments which were carried out at three different subphase pH values. From the use of the conventional Langmuir-Blodgett technique, the surface pressure isotherms, the rearrangement of the particles at the air-solution interphase under compressed and expansion, were quantified. In addition, the movement of particles in the monolayer at a range of surface pressures was measured and the packing factor of the interfacial particles was determined. The particles were observed to initially form rafts which closed up and rearranged as the surface pressure increased and more hydrophobic particles (less densely charged) produced more closely packed rafts. It was also found that as the surface pressure on the film increased, the average separation between rafts decreased and the percentage of voids in the network decreased resulting in a higher packing factor. The interaction forces, the cohesive pressure and the capillary force interactions were calculated for two particle submerged particles and related to the experimental data.

From the modified Langmuir-Blodgett trough, the influence of the particle flow under different rates of compression and on the packing factor as the bubble approached the Langmuir-Blodgett film was studied, together with the influence of pH and hydrophobicity on particle displacement during compression. Two distinct types of behavior occurred involving the rotation and the clustering of particles which caused the filling of interfacial voids. As the surface pressure on the film increased, the average separation distance decreased and the percentage of voids in the network decreased, resulting in a higher packing factor. The correlation coefficient data could be used to explain the differences in the direction in which the particles flow and also the collapse of the voids. Video recording showed that the packing factor significantly affected the movement of particles in the less densely packed regions. Finally, the kinetics of coalescence of the bubble with the Langmuir-Blodgett film was studied by recording the velocity of the ripples which spread out from the contact region. In addition, the healing of the interfacial layer was measured. It was found that the speed of the ripple was considerably reduced by the presence of particles in the film indicating the particles play an important role in controlling the response of the interface to surface perturbations.

## Supporting document

The re-arranging of particles at the interface—rotation of a particle group (AVI), The re-arranging of particles at the interface—void collapse (AVI), The spreading ripple on a particle-laden interface (AVI), The code for fitting data with Cubic Spline method (DOC) Supplementary Data Sheet 1.

## Author contributions

XY data collection, data analysis and interpretation and drafting the article. AM data collection regarding the particle tracking. RP helped to evaluate and edit the manuscript. GB and SA conception or design of the work; critical revision of the article and final approval of the version to be published.

### Conflict of interest statement

The handling editor declared a past co-authorship with one of the authors SA. The remaining authors declare that the research was conducted in the absence of any commercial or financial relationships that could be construed as a potential conflict of interest.

## Supplementary material

Movie S1 The re-arranging of particles at the interface.

Movie S2 The re-arranging of particles at the interface-void collapse.

Movie S3 The spreading ripple on a particle-laden interface.

Supplementary Data Sheet 1 The code for fitting data with Cubic Spline method (DOC).
