# Erratum: Experimental Technique to Study the Interaction Between a Bubble and the Particle-Laden Interface

**DOI:** 10.3389/fchem.2018.00474

**Published:** 2018-10-09

**Authors:** Frontiers Production Office

**Affiliations:** Frontiers Production Office, Frontiers Media SA, Switzerland

**Keywords:** particle monolayer, packing factor, particle tracking, surface pressure, bubble coalescence

Due to a production error, the first sentence of the conflict of interest statement was inadvertently not included. The complete statement should read:

The handling editor declared a past co-authorship with one of the authors SA.

The remaining authors declare that the research was conducted in the absence of any commercial or financial relationships that could be construed as a potential conflict of interest.

The publisher apologizes for this mistake.

The original article has been updated.

