# Supplementary material for: Experimental Technique to Study the Interaction Between a Bubble and the Particle-Laden Interface
Source: Front Chem. 2018 Aug 14;6:348. doi: 10.3389/fchem.2018.00348 (PMC6102402; doi:10.3389/fchem.2018.00348)
Supplement: Supplementary Data Sheet 1 — The code for fitting data with Cubic Spline method (DOC). [file Data_Sheet_1.DOCX]

**Experimental technique to study the interaction between a bubble and the particle-laden interface**

Xingshi Yang ^1^, Alexander Mayer ^1^, Ghislain Bournival ^1^, Robert Pugh ^2^, Seher Ata ^1*^

^1^ School of Mining Engineering, University of New South Wales, Sydney, NSW, 2052, Australia

^2^ Department of Physics and Mathematics, Nottingham Trent University, Nottingham, Nottinghamshire, United Kingdom

^*^ Corresponding author: s.ata@unsw.edu.au

**Supporting information**

Code for fitting data with Cubic Spline:

import pandas as pd

import matplotlib.pyplot as plt

import numpy as np

import matplotlib.ticker

from scipy import interpolate

df=pd.read_excel('F:\\filename.xlsx', 'sheetname')

fig, ax = plt.subplots(1, 1)

ax.plot(df['colume1'], df['data'], linestyle='-',linewidth='1.5',label='PL in $D_2O$')

x=df['colume1']

y=df['data']

tck = interpolate.splrep(x, y,k=4,s=10000)

y_int = interpolate.splev(x, tck, der = 0)

fig = plt.figure(figsize = (5.15,5.15))

plt.subplot(111)

plt.plot(x, y, marker = 'o', linestyle='',color='green')

plt.plot(x, y_int, linestyle = '-', linewidth = 1.5, color='red')

plt.show()

df2 = pd.DataFrame(y_int)

df2.to_csv("file_path.csv")
